# Supplementary material for: Oligogenic heterozygous inheritance of sperm abnormalities in mouse
Source: eLife. 2022 Apr 22;11:e75373. doi: 10.7554/eLife.75373 (PMC9071268; doi:10.7554/eLife.75373)
Supplement: Figure 5—source data 2. — DoF = Degrees of Freedom; CI = Confidence Interval. [file elife-75373-fig5-data2.docx]

**Figure 5- source data 2.** Statistical data associated to the Student *t*-test performed in Figure 5D-F. DoF = Degrees of Freedom ; CI = Confidence Interval.

|  | **Motility** | | | | **VAP** | | | | **VSL** | | | |
| --- | --- | --- | --- | --- | --- | --- | --- | --- | --- | --- | --- | --- |
| **Groups** | **t-value** | **DoF** | **p-value** | **95% CI** | **t-value** | **DoF** | **p-value** | **95% CI** | **t-value** | **DoF** | **p-value** | **95% CI** |
| Wild-type vs  Cfap44 | 0.73583 | 20.322 | 0.4702 | -6.024568 /  12.601657 | 0.33461 | 22.983 | 0.741 | -17.29417 /  23.96806 | 0.83871 | 23 | 0.4103 | -10.82677 /  25.59249 |
| Wild-type vs  Cfap44/Cfap43 | 0.93212 | 21.077 | 0.3618 | -6.334437 /  16.629709 | 3.9479 | 21.665 | 0.000701 | 19.51192 /  62.77780 | 4.2279 | 21.881 | 0.0003492 | 19.13081 /  55.99091 |
| Wild-type vs  Cfap44/Cfap43/Armc2 | 0.12341 | 15.093 | 0.9034 | -9.881773 /  11.097045 | 3.744 | 16.842 | 0.001638 | 14.55987 /  52.21585 | 3.7382 | 16.606 | 0.001695 | 12.85173 /  46.29398 |
| Wild-type vs  Cfap44/Cfap43/Armc2/Ccdc146 | 1.4844 | 4.7854 | 0.2004 | -7.111589 /  25.940194 | 4.5767 | 6.1886 | 0.003498 | 23.99476 /  78.26762 | 5.0332 | 6.5295 | 0.001853 | 25.16275 /  71.01630 |
| Cfap44 vs  Cfap44/Cfap43 | 0.40858 | 14.443 | 0.6888 | -7.87191 /  11.59009 | 3.9209 | 18.575 | 0.000951 | 17.59425 /  58.02157 | 3.6364 | 18.898 | 0.001769 | 12.80207 /  47.55393 |
| Cfap44 vs  Cfap44/Cfap43/Armc2 | -0.70525 | 8.5243 | 0.4995 | -11.353847 /  5.992028 | 3.7596 | 13.771 | 0.00217 | 12.88052 /  47.22130 | 3.0616 | 13.649 | 0.008665 | 6.607495 /  37.772505 |
| Cfap44 vs  Cfap44/Cfap43/Armc2/Ccdc146 | 1.1105 | 2.861 | 0.3513 | -11.92255 /  24.17406 | 4.5741 | 4.8889 | 0.006319 | 20.74965 /  74.83884 | 4.5165 | 5.3105 | 0.005445 | 17.94003 /  63.47331 |
| Cfap44/Cfap43 vs  Cfap44/Cfap43/Armc2 | -0.90847 | 12.731 | 0.3805 | -15.35956 /  6.27956 | -0.90759 | 12.997 | 0.3806 | -26.22173 /  10.70773 | -1.0873 | 12.899 | 0.2968 | -23.871489 /  7.895489 |
| Cfap44/Cfap43 vs  Cfap44/Cfap43/Armc2/Ccdc146 | 0.66671 | 4.8174 | 0.5356 | -12.37333 /  20.90666 | 0.918 | 5.4867 | 0.3972 | -17.24767 /  37.22033 | 1.158 | 5.3976 | 0.2955 | -12.33536 /  33.39269 |
| Cfap44/Cfap43/Armc2 vs  Cfap44/Cfap43/Armc2/Ccdc146 | 1.495 | 3.5109 | 0.2188 | -8.487843 /  26.101176 | 1.8782 | 3.342 | 0.1475 | -10.65390 /  46.14057 | 2.2736 | 3.6141 | 0.0925 | -5.08036 /  42.11369 |
