# Supplementary material for: Oligogenic heterozygous inheritance of sperm abnormalities in mouse
Source: eLife. 2022 Apr 22;11:e75373. doi: 10.7554/eLife.75373 (PMC9071268; doi:10.7554/eLife.75373)
Supplement: Figure 5—source data 3. — DoF = Degrees of Freedom; CI = Confidence Interval. [file elife-75373-fig5-data3.docx]

**Figure 5- source data 3.** Statistical data associated to the Student *t*-test performed in Figure 5A-C. DoF = Degrees of Freedom ; CI = Confidence Interval.

|  | **Abnormal cells** | | | | **Head anomalies** | | | | **Flagellum anomalies** | | | |
| --- | --- | --- | --- | --- | --- | --- | --- | --- | --- | --- | --- | --- |
| **Groups** | **t-value** | **DoF** | **p-value** | **95% CI** | **t-value** | **DoF** | **p-value** | **95% CI** | **t-value** | **DoF** | **p-value** | **95% CI** |
| Wild-type vs  Cfap44 | -10.152 | 11.224 | 0.0000005 | -17.08217 / -11.00712 | -7.2605 | 8.2481 | 7.467e-05 | -16.555175 / -8.605539 | -2.8342 | 7.2675 | 0.02428 | -6.1699235 / -0.5800765 |
| Wild-type vs  Cfap44/Cfap43 | -10.533 | 12.33 | 0.0000001 | -24.23028 / -15.94472 | -11.822 | 14.527 | 7.564e-09 | -19.52769 / -13.54731 | -3.1518 | 10.163 | 0.0101 | -7.972725 / -1.377275 |
| Wild-type vs  Cfap44/Cfap43/Armc2 | -10.134 | 3.7705 | 0.0007164 | -29.37527 / -16.49973 | -5.2884 | 3.1659 | 0.0115 | -34.756351 / -9.118649 | -2.2486 | 3.1584 | 0.1056 | -12.176935 / 1.926935 |
| Wild-type vs  Cfap44/Cfap43/Armc2/Ccdc146 | -9.9553 | 2.2268 | 0.006873 | -45.28961 / -19.75205 | -11.112 | 2.2134 | 0.005525 | -45.71007 / -21.83160 | -2.7878 | 2.4134 | 0.08774 | -7.81574 / 1.06574 |
| Cfap44 vs  Cfap44/Cfap43 | -2.8709 | 14.463 | 0.01201 | -10.543812 / -1.541902 | -1.968 | 12.305 | 0.07202 | -8.326176 / 0.411890 | -0.7099 | 14.983 | 0.4887 | -5.203617 / 2.603617 |
| Cfap44 vs  Cfap44/Cfap43/Armc2 | -3.6561 | 4.8317 | 0.01556 | -15.211449 / -2.574266 | -2.1302 | 3.9352 | 0.1013 | -21.632363 / 2.918077 | -0.69456 | 4.5699 | 0.521 | -8.414446 / 4.914446 |
| Cfap44 vs  Cfap44/Cfap43/Armc2/Ccdc146 | -5.4567 | 2.5561 | 0.01812 | -30.391859 / -6.560522 | -6.2977 | 3.2368 | 0.006426 | -31.46904 / -10.91191 | 0 | 5.8909 | 1 | -3.977791 / 3.977791 |
| Cfap44/Cfap43 vs  Cfap44/Cfap43/Armc2 | -1.0312 | 7.3032 | 0.3354 | -9.330988 / 3.630988 | -1.2641 | 3.5515 | 0.2828 | -17.875401 / 7.075401 | -0.16852 | 5.6377 | 0.8721 | -7.087038 / 6.187038 |
| Cfap44/Cfap43 vs  Cfap44/Cfap43/Armc2/Ccdc146 | -3.4237 | 3.3337 | 0.03548 | -23.362661 / -1.504006 | -5.3758 | 2.7237 | 0.01608 | -28.046580 / 40.33333 | 0.70491 | 8.4808 | 0.4998 | -2.911151 / 5.511151 |
| Cfap44/Cfap43/Armc2 vs  Cfap44/Cfap43/Armc2/Ccdc146 | -2.5018 | 3.7087 | 0.07153 | -20.556468 / 1.389802 | -2.3421 | 4.9352 | 0.06689 | -24.87269 / 1.20602 | 0.69197 | 4.3371 | 0.5242 | -5.061554 / 8.561554 |
