# Supplementary material for: Oligogenic heterozygous inheritance of sperm abnormalities in mouse
Source: eLife. 2022 Apr 22;11:e75373. doi: 10.7554/eLife.75373 (PMC9071268; doi:10.7554/eLife.75373)
Supplement: Figure 6—source data 2. — DoF = Degrees of Freedom; CI = Confidence Interval. [file elife-75373-fig6-data2.docx]

**Figure 6-source data 2.** Statistical data associated to the Student *t*-test performed in figure 6D-F. DoF = Degrees of Freedom ; CI = Confidence Interval.

|  | **Motility** | | | | **VAP** | | | | **VSL** | | | |
| --- | --- | --- | --- | --- | --- | --- | --- | --- | --- | --- | --- | --- |
| **Groups** | **t-value** | **DoF** | **p-value** | **95% CI** | **t-value** | **DoF** | **p-value** | **95% CI** | **t-value** | **DoF** | **p-value** | **95% CI** |
| Wild-type vs One gene | 0.841 | 24.641 | 0.4084 | -5.630928 / 13.393941 | 0.0452 | 23.652 | 0.9643 | -18.09020 / 18.90011 | 0.5068 | 24.877 | 0.6167 | -12.33232 / 20.38062 |
| Wild-type vs Two genes | 1.0391 | 21.898 | 0.3101 | -4.615605 / 13.880107 | 4.4772 | 21.248 | 0.0002025 | 20.82954 / 56.91617 | 4.5621 | 21.809 | 0.0001558 | 18.98931 / 50.67333 |
| Wild-type vs Three genes | 0.8178 | 19.972 | 0.4231 | -5.987244 / 13.707515 | 4.5572 | 19.907 | 0.0001935 | 22.68524 / 61.00297 | 4.5906 | 19.999 | 0.0001772 | 20.49769 / 54.64053 |
| Wild-type vs Four genes | 1.4844 | 4.785 | 0.2004 | -7.111589 / 25.940194 | 4.5767 | 6.189 | 0.003498 | 23.99476 / 78.26762 | 5.0332 | 6.529 | 0.001853 | 25.16275 / 71.01630 |
| One gene vs Two genes | 0.2223 | 54.935 | 0.8249 | -6.017897 / 7.519386 | 6.0466 | 54.958 | 1.356e-07 | 25.71811 / 51.21770 | 5.2996 | 54.864 | 2.122e-06 | 19.15681 / 42.45753 |
| One gene vs Three genes | -0.0057 | 20.765 | 0.9955 | -7.756221 / 7.713479 | 5.8961 | 20.437 | 8.347e-06 | 26.79846 / 56.07984 | 5.147 | 20.202 | 4.76e-05 | 19.95870 / 47.13122 |
| One gene vs Four genes | 0.9818 | 3.128 | 0.3959 | -11.99422 / 23.05982 | 5.3499 | 3.546 | 0.008223 | 23.01485 / 78.43762 | 5.3921 | 3.908 | 0.006112 | 21.16409 / 66.96666 |
| Two genes vs Three genes | -0.2194 | 17.41 | 0.8289 | -8.184485 / 6.640255 | 0.4449 | 17.291 | 0.6619 | -11.10106 / 17.04356 | 0.4457 | 16.674 | 0.6615 | -10.24067 / 15.71625 |
| Two genes vs Four genes | 0.8683 | 2.856 | 0.452 | -13.25433 / 22.81843 | 1.3287 | 3.184 | 0.2711 | -16.16664 / 40.68331 | 1.6833 | 3.384 | 0.1805 | -10.27309 / 36.78950 |
| Three genes vs Four genes | 0.9707 | 3.259 | 0.3981 | -11.86376 / 22.97209 | 0.9577 | 3.739 | 0.3959 | -18.39442 / 36.96859 | 1.251 | 4.148 | 0.2769 | -12.50366 / 33.54450 |
