# Supplementary material for: Oligogenic heterozygous inheritance of sperm abnormalities in mouse
Source: eLife. 2022 Apr 22;11:e75373. doi: 10.7554/eLife.75373 (PMC9071268; doi:10.7554/eLife.75373)
Supplement: Figure 6—source data 3. — DoF = Degrees of Freedom; CI = Confidence Interval. [file elife-75373-fig6-data3.docx]

**Figure 6- source data 3.** Statistical data associated to the Student *t*-test performed in figure 6A-C. DoF = Degrees of Freedom ; CI = Confidence Interval.

|  | **Abnormal cells** | | | | **Head anomalies** | | | | **Flagellum anomalies** | | | |
| --- | --- | --- | --- | --- | --- | --- | --- | --- | --- | --- | --- | --- |
| **Groups** | **t-value** | **DoF** | **p-value** | **95% CI** | **t-value** | **DoF** | **p-value** | **95% CI** | **t-value** | **DoF** | **p-value** | **95% CI** |
| Wild-type vs One gene | -3.1701 | 29.521 | 0.003537 | -9.055057 /  -1.956307 | -2.1801 | 28.422 | 0.03768 | -7.105830 /  -0.223715 | -3.4797 | 31.422 | 0.001495 | -4.198744 /  -1.096710 |
| Wild-type vs Two genes | -7.7418 | 24.187 | 5.324e-08 | -18.70144 /  -10.83145 | -7.1862 | 24.8 | 1.644e-07 | -15.258544 /  -8.458561 | -4.1232 | 24.277 | 0.0003785 | -5.695024 /  -1.897081 |
| Wild-type vs Three genes | -9.5403 | 5.023 | 0.0002089 | -27.39619 /  -15.77881 | -6.2533 | 4.342 | 0.002524 | -30.09462 /  -11.98038 | -1.6895 | 4.22 | 0.1626 | -9.853102 /  2.303102 |
| Wild-type vs Four genes | -9.9553 | 2.227 | 0.006873 | -45.28961 /  -19.75205 | -11.1123 | 2.213 | 0.005525 | -45.71007 /  -21.83160 | -2.7878 | 2.413 | 0.08774 | -7.81574 /  1.06574 |
| One gene vs Two genes | -3.9366 | 37.644 | 0.0003443 | -14.02456 /  -4.49697 | -3.8069 | 38.883 | 0.0004871 | -12.547769 /  -3.839791 | -1.0653 | 35.6 | 0.2939 | -3.335357 /  1.038706 |
| One gene vs Three genes | -6.0713 | 8.974 | 0.0001878 | -22.07652 /  -10.08711 | -4.7767 | 5.881 | 0.003243 | -26.315938 /  -8.429517 | -0.4893 | 4.761 | 0.6463 | -7.139300 /  4.884755 |
| One gene vs Four genes | -7.6205 | 3.072 | 0.004313 | -38.14805 /  -15.88225 | -9.0179 | 3.202 | 0.002213 | -40.36086 /  -19.85126 | -0.5451 | 3.527 | 0.6183 | -4.636018 /  3.181472 |
| Two genes vs Three genes | -2.4681 | 10.19 | 0.0328 | -12.9633305 /  -0.6787747 | -2.5336 | 5.787 | 0.04589 | -18.1235924 /  -0.2343023 | 0.0089 | 5.239 | 0.9932 | -5.966318 /  6.008423 |
| Two genes vs Four genes | -4.8888 | 3.368 | 0.01243 | -28.628966 /  -6.879806 | -6.594 | 3.142 | 0.006141 | -32.22232 /  -11.60224 | 0.2942 | 4.575 | 0.7815 | -3.363397 /  4.205502 |
| Three genes vs Four genes | -2.8544 | 3.822 | 0.04873 | -21.7660625 /  -0.1006041 | -2.8734 | 5.67 | 0.03017 | -23.731335 /  -1.735332 | 0.1607 | 5.646 | 0.8779 | -5.783259 /  6.583259 |
