# Supplementary material for: Oligogenic heterozygous inheritance of sperm abnormalities in mouse
Source: eLife. 2022 Apr 22;11:e75373. doi: 10.7554/eLife.75373 (PMC9071268; doi:10.7554/eLife.75373)
Supplement: Figure 7—source data 2. — DoF = Degrees of Freedom; CI = Confidence Interval. [file elife-75373-fig7-data2.docx]

**Figure 7- source data 2.** Statistical data associated to the Student *t*-test performed in Figure 7B. DoF = Degrees of Freedom ; CI = Confidence Interval.

|  | **Time between litters** | | | | **Pups per litter** | | | |
| --- | --- | --- | --- | --- | --- | --- | --- | --- |
| **Groups** | **t-value** | **DoF** | **p-value** | **95% CI** | **t-value** | **DoF** | **p-value** | **95% CI** |
| Wild-type vs One gene | 0.799 | 28.297 | 0.4309 | -3.329117 / 7.590481 | 0.8831 | 49.559 | 0.3814 | -0.8051491 / 2.0681794 |
| Wild-type vs Two genes | 0.5176 | 12.019 | 0.6141 | -6.502971 / 10.556542 | -0.307 | 20.747 | 0.7619 | -1.822674 / 1.354103 |
| Wild-type vs Three genes | 0.1424 | 8.816 | 0.89 | -11.07293 / 12.55507 | 1.4973 | 16.026 | 0.1538 | -0.6151817 / 3.5751817 |
| Wild-type vs Four genes | 0.7095 | 25.988 | 0.4843 | -3.913203 / 8.038203 | 0.8097 | 34.683 | 0.4236 | -0.8494933 / 1.9761600 |
| One gene vs Two genes | -0.0294 | 8.672 | 0.9772 | -8.152654 / 7.944862 | -1.2588 | 16.454 | 0.2257 | -2.3206518 / 0.5890501 |
| One gene vs Three genes | -0.282 | 7.207 | 0.7859 | -12.97425 / 10.19503 | 0.9109 | 13.25 | 0.3786 | -1.160005 / 2.856975 |
| One gene vs Four genes | -0.0287 | 25.404 | 0.9773 | -4.952527 / 4.816163 | -0.1114 | 35.06 | 0.9119 | -1.310622 / 1.174259 |
| Two genes vs Three genes | -0.2255 | 10.609 | 0.8259 | -13.89055 / 11.31912 | 1.7693 | 12.98 | 0.1003 | -0.3792307 / 3.8078021 |
| Two genes vs Four genes | 0.0096 | 10.092 | 0.9925 | -8.247438 / 8.318867 | 1.1947 | 13.174 | 0.2533 | -0.642762 / 2.238000 |
| Three genes vs Four genes | 0.2611 | 7.932 | 0.8007 | -10.36885 / 13.01170 | -1 | 12.112 | 0.3369 | -2.911868 / 1.078535 |
