# Supplementary material for: Oligogenic heterozygous inheritance of sperm abnormalities in mouse
Source: eLife. 2022 Apr 22;11:e75373. doi: 10.7554/eLife.75373 (PMC9071268; doi:10.7554/eLife.75373)
Supplement: Figure 7—source data 3. — DoF = Degrees of Freedom; CI = Confidence Interval. [file elife-75373-fig7-data3.docx]

**Figure 7- source data 3.** Statistical data associated to the Student *t*-test performed in Figure 7A. DoF = Degrees of Freedom ; CI = Confidence Interval.

|  | **Testis weight** | | | | **Concentration** | | | |
| --- | --- | --- | --- | --- | --- | --- | --- | --- |
| **Groups** | **t-value** | **DoF** | **p-value** | **95% CI** | **t-value** | **DoF** | **p-value** | **95% CI** |
| Wild-type vs One gene | 0.3325 | 17.949 | 0.7433 | -0.008462764 / 0.011644907 | 0.446 | 33.073 | 0.6585 | -3.540883 / 5.529633 |
| Wild-type vs Two genes | -1.1798 | 31.898 | 0.2468 | -0.013589384 / 0.003622076 | -0.3982 | 22.979 | 0.6942 | -4.863821 / 3.293629 |
| Wild-type vs Three genes | -1.6502 | 9.077 | 0.133 | -0.022387499 / 0.003487499 | -0.3099 | 13.934 | 0.7612 | -7.527883 / 5.627883 |
| Wild-type vs Four genes | -0.7729 | 2.284 | 0.5116 | -0.03752964 / 0.02492131 | -0.8913 | 5.69 | 0.4089 | -9.037768 / 4.258601 |
| One gene vs Two genes | -1.1606 | 30.436 | 0.2548 | -0.018137549 / 0.004988099 | -1.0824 | 51.51 | 0.2841 | -5.079069 / 1.520126 |
| One gene vs Three genes | -1.6098 | 15.548 | 0.1276 | -0.025615301 / 0.003533158 | -0.6785 | 11.586 | 0.5108 | -8.213397 / 4.324647 |
| One gene vs Four genes | -0.8784 | 3.327 | 0.4386 | -0.03497451 / 0.01918404 | -1.3811 | 4.208 | 0.2361 | -10.056445 / 3.288528 |
| Two genes vs Three genes | -0.6892 | 14.324 | 0.5017 | -0.018335923 / 0.009403231 | -0.0618 | 8.868 | 0.9521 | -6.219170 / 5.889363 |
| Two genes vs Four genes | -0.1517 | 2.953 | 0.8892 | -0.02926857 / 0.02662754 | -0.7233 | 2.857 | 0.5242 | -8.868781 / 5.659806 |
| Three genes vs Four genes | 0.3303 | 4.009 | 0.7577 | -0.02326937 / 0.02956104 | -0.4457 | 7.686 | 0.6681 | -8.940743 / 6.061576 |
